# Exploring the sustainable energy Potential of Positive Energy Districts (PED) via Geographic Information System (GIS)

**DOI:** 10.12688/openreseurope.19075.1

**Published:** 2025-03-26

**Authors:** Hande Demirel, Ayşenur Koçyiğit, Ben Alpagut, Damla Muhcu

**Affiliations:** 1 Faculty of Civil Engineering, Department of Geomatics Engineering, Istanbul Technical University, İstanbul, 34469, Turkey; 2 Faculty of Civil Engineering, Department of Geomatics Engineering, Istanbul Technical University, İstanbul, 34469, Turkey; 3 Smart and Sustainable Cities Department, Demir Energy, İstanbul, 34718, Turkey; 4 Department of Project Management, Municipality of Kadıköy, İstanbul, 34716, Turkey

**Keywords:** Positive Energy District (PED), Renewable Energy, Geographic Information Systems (GIS), Solar Energy, Smart Cities, Sustainability

## Abstract

**Background:**

The Positive Energy District (PED) concept aims to transform urban areas into carbon-neutral and zero-energy communities by reducing energy consumption and increasing renewable energy production. However, challenges persist in facilitating effective decision-making. GIS-based frameworks offer mature solutions to help overcome these issues.

**Methods:**

This study describes a GIS-based framework to integrate databases, conduct spatial analyses, and support decision-making through spatiotemporal analyses of solar energy potential to support decision-making in PEDs. Furthermore, the developed concepts are deployed in the case study area – the Caferağa neighborhood of Kadıköy, Türkiye.

**Results:**

According to the results of spatial analyses including sunlight exposure and shading effects, there is significant potential for solar energy within the district, especially in spring and summer seasons. The total solar energy was 629 million kWh, with an average solar energy of 1,410 kWh/m². Three-dimensional (3D) spatial analyses indicate that the solar energy potential is high on both roofs and various facades of the building.

**Conclusion:**

The achieved results are promising, where co-creation, co-design- and co-implementation could be performed collaboratively via designed platform that seamlessly integrate data, functions, necessary tools and expertise.

## Introduction

The primary goal of the Positive Energy District (PED) is to generate more energy than the district consumes, thus creating a surplus through renewable technologies. In essence, PEDs strive to establish sustainable communities that rely entirely on renewable energy sources. However, it is a challenge for several districts world-wide, since it is vital for new buildings constructed in the area to meet energy efficiency targets, where, existing buildings must be transformed and improved to enhance their energy efficiency ( Jepsen *et al.*, 2022). Furthermore, PEDs are expected to not only produce enough energy to meet citizens’ needs but also generate more local renewable energy than is consumed outside the region. In short, there will be an excess of energy while maintaining a net zero energy balance (Davis, K. (2009). Towards an ICT Infrastructure for Energy Positive Neighbourhoods: Report from ELSA Thematic Working Group on ICT for Energy Efficiency. http://ec.europa.eu/information_society/activities/sustainable_growth/docs/elsa/elsa_1/ELSA-EnergyPositive-Report1.pdf), which is a challenge in itself.

The PED concept has very strong spatial pillar, where Geographic Information Science (GIS) is a mature ecosystem for successfully managing such challenges. Especially, decision making for planning, managing and operation such systems requires support from spatial analyses and multi-criteria decision making. Furthermore, via such ecosystems, visualization of PEDs to various stake-holders including citizens could be successfully performed ( Alpagut *et al.*, 2021). There are several recent studies that integrate PED and GIS successfully ( Alpagut *et al.*, 2019; Lindholm *et al.*, 2021; Zhang *et al.*, 2021). However, there are still research questions that need to be addressed in the context of PEDs. Some of them are listed below and further analyzed within the scope of this manuscript.

PED is a concept that emphasizes the importance of active interaction between energy production systems, energy consumers and energy storage within a region ( Lindholm *et al.*, 2021). The concept of PED results from an extensive co-creation process and incorporates various multidimensional features, aiming to foster sustainable development in urban areas and the transition of cities towards a climate-neutral energy system ( Turci *et al.*, 2022). Several factors including renewable energy sources, energy storage capacity, financial aspects, energy consumption patterns, and population have an effect on PEDs, where their relative importance should be assessed. Furthermore, recent interest and developments in micro-mobility, including electric vehicles, could contribute to PEDs as potential consumers. Several research questions remain, such as the location selection of charging stations, energy storage, and their degree of contribution to the PED ( Zhang *et al.*, 2021).

Even though bidirectional grids are expanding, electricity remains the primary mode of transmission in most buildings. Incorporating electric vehicles (EVs), particularly in vehicle-to-grid (V2G) applications, introduces another significant aspect; however, the most crucial factor remains the building itself, which must effectively address the needs of its users while reducing energy consumption and environmental impact. ( Ala-Juusela *et al.*, 2021). Hence, energy-efficient solutions require more data-driven management and control strategies. 

PED studies are carried out to retrofit residential buildings to reduce building energy consumption in order to maximize infrastructure performance. This can involve measures such as high-performance insulation, efficient windows, heat recovery systems, smart thermostats with real-time energy consumption sensors, and advanced energy management systems as implemented in Groningen North and Groningen South within the MAKING-CITY project ( Velez & Sanz, 2019). Such challenges can be addressed through digital twins, where monitoring and scenario testing are vital. To achieve this goal, 3D models such as CityGML and Building Information Modelling (BIM) could be utilized and coupled with various sensors. Some important European Union Horizon 2020 PED projects, such as MAKING-CITY, CityxChange, and POCITYF, are introduced below in Table 1 as pioneers on this topic (ATELIER. (2024). AmsTErdam BiLbao cItizen drivEn smaRt cities. https://smartcity-atelier.eu/; CityxChange. (n.d.). CityxChange. https://cityxchange.eu/; MAKING-CITY. (2019). Making City. https://makingcity.eu/; POCITYF. (n.d.). POCITYF. https://pocityf.eu/; Stardust. (n.d.). STARDUST: Enlightening European Cities. https://stardustproject.eu/).

**Table 1.** Horizon 2020 PED Projects ATELIER. (2024). AmsTErdam BiLbao cItizen drivEn smaRt cities. https://smartcity-atelier.eu/; CityxChange. (n.d.). CityxChange. https://cityxchange.eu; MAKING-CITY. (2019). Making City. https://makingcity.eu/; POCITYF. (n.d.). POCITYF. https://pocityf.eu/; Stardust. (n.d.). STARDUST: Enlightening European Cities. https://stardustproject.eu/).

Name	Scope	Date
MAKING-CITY	The aim of this project is to demonstrate the transformation of urban energy systems towards smart, low-carbon cities using the concept of PED.	2018–2024
STARDUST	STARDUST is a smart cities project focused on transforming cities that emit carbon into highly efficient, smart, and citizen-oriented urban environments. The project aims to develop innovative green solutions and business models while integrating building design, mobility, and energy efficiency.	2017–2024
CityxChange	The goal is to develop a framework and supporting tools to enable a unified energy market, foster a connected community, and provide recommendations for new policy interventions, market liberalization, and business models. These initiatives aim to support positive energy communities and integrate e-Mobility as a Service (eMaaS).	2018–2023
POCITYF	POCITYF is a smart city project designed to help historical cities become greener, smarter, and more livable while preserving their cultural heritage.	2019–2024
ATELIER	The project aims to enhance the local innovation ecosystem by conducting PED Innovation Workshops, which will help eliminate legal, financial, and social barriers to implementing smart solutions. These workshops will become self-sustaining after the project concludes, serving as engines for scaling and replicating solutions in ATELIER cities and beyond.	2019–2024

These projects address various aspects of PED challenges. MAKING-CITY aims to enhance citizen engagement by developing robust city visions and facilitating knowledge sharing to create district energy plans and PEDs through a bottom-up approach. ATELIER focuses on establishing smart grids within a community-owned framework, enabling the sharing, distribution, and trading of renewable energy among individuals and organizations. POCITYF has introduced peer-to-peer (P2P) energy trading platforms, empowering citizens to take charge of energy resilience and actively engage in decision-making processes ( Olivadese *et al.*, 2021). CityxChange has developed a toolbox to design, analyze, and manage the local energy system's grid operations, including storage and grid balancing. The tools are designed to recommend the most cost-effective design for a district aiming to become a Positive Energy Block (PEB). The calculations provide accurate information about the local grid topology for day-ahead operations, estimate generation and load at each connection point, and determine exactly how local resources will affect the local grid ( Dahlen *et al.*, 2020). These projects provide innovative solutions that integrate physical investments with social engagement in the energy transition. They enhance collaboration among traditional and emerging stakeholders involved in urban energy transitions by using integrated participatory processes. In this context, developing multi-scale and multi-level engagement strategies, legitimizing public initiatives to promote the public good of energy citizenship, and presenting quantitative evidence on the benefits of integrated planning processes emphasize the potential of a unified approach, where several cities especially in Europe are looking for ( Olivadese *et al.*, 2021).

Another recent important program funded by JPI Urban Europe, namely Positive Energy Regions and Cities (ENPED), is designed to enhance the research and innovation capacity of the European Union (EU) in pursuit of climate neutrality across 100 regions by 2030. One of the projects funded under this program being the Positive Robust PED Localities (PROPEL), where authors are also contributing, aims to promote the utilization of advanced energy carriers within the food system, while also advancing the sustainable management of waste generated along transportation networks through biogas energy. By doing so, PROPEL intends to expand off-grid operational capabilities and facilitate the development of PEDs. Within these projects, mainly a GIS-based decision support systems are required and developed to ensure the collaborative environment that is essential and tools and analyses are also deployed to facilitated and aid decision making process.

Several national PED projects benefit from spatial analyses to test cost-effectiveness of PEDs. For example, according to a study conducted in the Alfama district in Lisbon, the potential reduction in annual energy demand with building rehabilitation was 84% for space heating and 19% for cooling. ( Gouveia *et al.*, 2021). At the same time, the integration of building-integrated Photovoltaics (PV) technologies on roofs and windows potentially provides production up to 60 GWh/year. When evaluated on a regional scale, these two components of the PED concept can require an investment of 60M€ to 81M€ depending on the PV technologies on roofs, which is a challenging aspect in historic districts ( Gouveia *et al.*, 2021). PEDs do not necessarily have to be an isolated energy islands; they should be integrated into larger energy system. Since there will be more or less intense overproduction (e.g., solar on a spring day) and consumption peaks (e.g., on a very cold and dark winter evening), developers should be informed and take these into account ( Shnapp *et al.*, 2020).

Among these challenges, this study focuses on the research questions outlined below.

-   What are the factors influencing energy production from renewable sources, particularly solar energy? How can this be analyzed?

-   What types of data–both spatial and non-spatial–are necessary for conducting such analyses? How could data quality affect the results obtained?

A spatial PED framework has been developed and tested to address key questions using an extensive database and spatial analysis techniques. These concepts have been deployed in the Caferağa neighborhood of Kadıköy, Türkiye. The study includes assessments of sunlight exposure, shading effects, and the overall solar energy potential within the Caferağa neighborhood. The sunlight analysis quantifies how many hours different facades and surrounding areas of the buildings in the study area are exposed to sunlight. It also examines how sunlight varies throughout the day in relation to shadow changes. Additionally, the solar potential analysis evaluates the solar capacity of the roofs and facades of the buildings. The research addresses several challenges, such as the availability and quality of data for spatial analysis, the interpretation of analytical findings, and the complexities of transforming a historic district into a PED.

## Methods

It is vital to customize PED to fit local contexts, while also recognizing that the core approach and methodology hold universal relevance. This study is situated in the Caferağa district of Istanbul, Turkey, where a range of noteworthy sustainability projects and initiatives are presently being undertaken. For example, the Sustainable Urban Mobility Plan (SUMP) developed by the Istanbul Metropolitan Municipality (IBB) designates the Caferağa Neighborhood as a low-emission zone. The plan aims to promote sustainable transportation solutions in the area. This initiative is a collaborative effort between IBB and the Kadıköy Municipality, focusing on reducing automobile use while improving accessibility for pedestrians and cyclists. Additionally, the Caferağa district serves as the pilot area for the MAKING-CITY project, which is one of the first Positive Energy District (PED) projects implemented in Türkiye.

Istanbul, located in the Marmara Region of Turkey, is the country's most populous city, boasting a population of 15,907,951 (Turkish Statistical Institute (TURKSTAT). (2022). Adrese Dayalı Nüfus Kayıt Sistemi Sonuçları. https://data.tuik.gov.tr/Bulten/Index?p=49685#:~:text=%C4%B0stanbul'un%20n%C3%BCfusu%2015%20milyon,907%20bin%20951%20ki%C5%9Fi%20oldu). Spanning an area of 5,712 km², Istanbul serves as a strategically significant metropolis that bridges the continents of Europe and Asia. Its prominence as a major trade center contributes to its high population and traffic density. The city is also renowned for its rich historical and cultural heritage, attracting numerous tourists to landmarks. Kadıköy, one of Istanbul's most developed districts, is situated on the Asian side and covers an area of 25.20 km². The district comprises 21 neighborhoods, making it an active area with both historical and modern structures. This district draws both locals and tourists alike during the day, owing to its array of cultural sites, historical buildings, restaurants, and cafes (Governor’s Office of Istanbul. (n.d.). Bir Bakışta İstanbul. http://www.istanbul.gov.tr/bir-bakista-istanbul; Municipality of Kadiköy. (n.d.). Coğrafi Konum. https://www.kadikoy.bel.tr/tr/cografi-konum) Caferağa Neighborhood is one of the oldest neighborhoods in Kadıköy. The neighborhood's total population is 22,763, and it is one of the neighborhoods with the highest daytime population in Kadıköy. The 55.8% of the population is female and 44.2% is male. The demographic distribution of the population indicates that 19.9% are aged between 0 and 24 years, 55.7% are aged between 25 and 59 years, and 27.4% are aged 60 years and above. 

**Figure 1.**
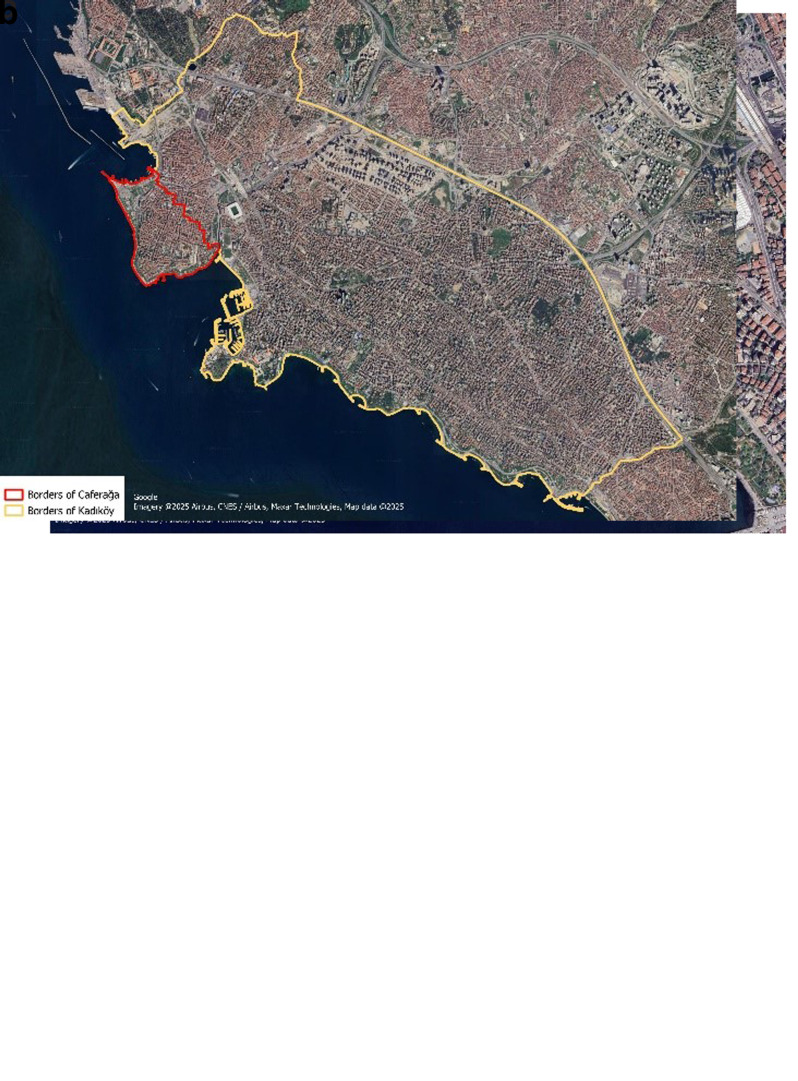
( **a**) Borders of Caferağa, ( **b**) Caferağa neighborhood in Kadıköy district (Google. (2022). Google Maps/Google Earth Additional Terms of Service. https://www.google.com/intl/en_tr/help/terms_maps/)

The total area of the neighborhood is 1,2374 km2, of which 0.028 km2 are residential areas, as illustrated in Figure 1. The area involves dense food and beverage shops, creative sectors, and entertainment, attracting several visitors and providing services on a metropolitan scale during day and night time. The economic viability of the neighborhood is quite high, with a Socio-Economic Status (SES) score of 100 in 2016 (Istanbul Metropolitan Municipality, Urban Planning Department. (n.d.). Caferağa Mahallesi. https://sehirplanlama.ibb.istanbul/caferaga-mahallesi/; The Istanbul Electricity, Tram and Tunnel. (IETT) (2024). IETT GTFS Data. https://data.ibb.gov.tr/en/dataset/iett-gtfs-verisi; Municipality of Kadiköy. (n.d.). Anlat Kadıköy. https://anlat.kadikoy.bel.tr/mahalleler/caferaga). The Caferağa Neighborhood contains 2,284 buildings constructed over various periods. Of these, 36.6% were built between 1908 and 1975, 32.8% between 1976 and 1987, and 30.6% between 1988 and 2019. Given that Istanbul is located in an earthquake zone, several of these buildings are undergoing transition and regeneration in accordance with the disaster-oriented Urban Regeneration Law ( CSB, 2018), where most of them remains vulnerable to disasters. Within the neighborhood, there are sea lines, metro stations, bus stops, and tram stops that offer transportation to other districts. Additionally, micro-mobility vehicles like scooters and bicycles are commonly used for transportation within and between districts. There are also 24 micro-mobility parking stations in the study area.

**Figure 2.**
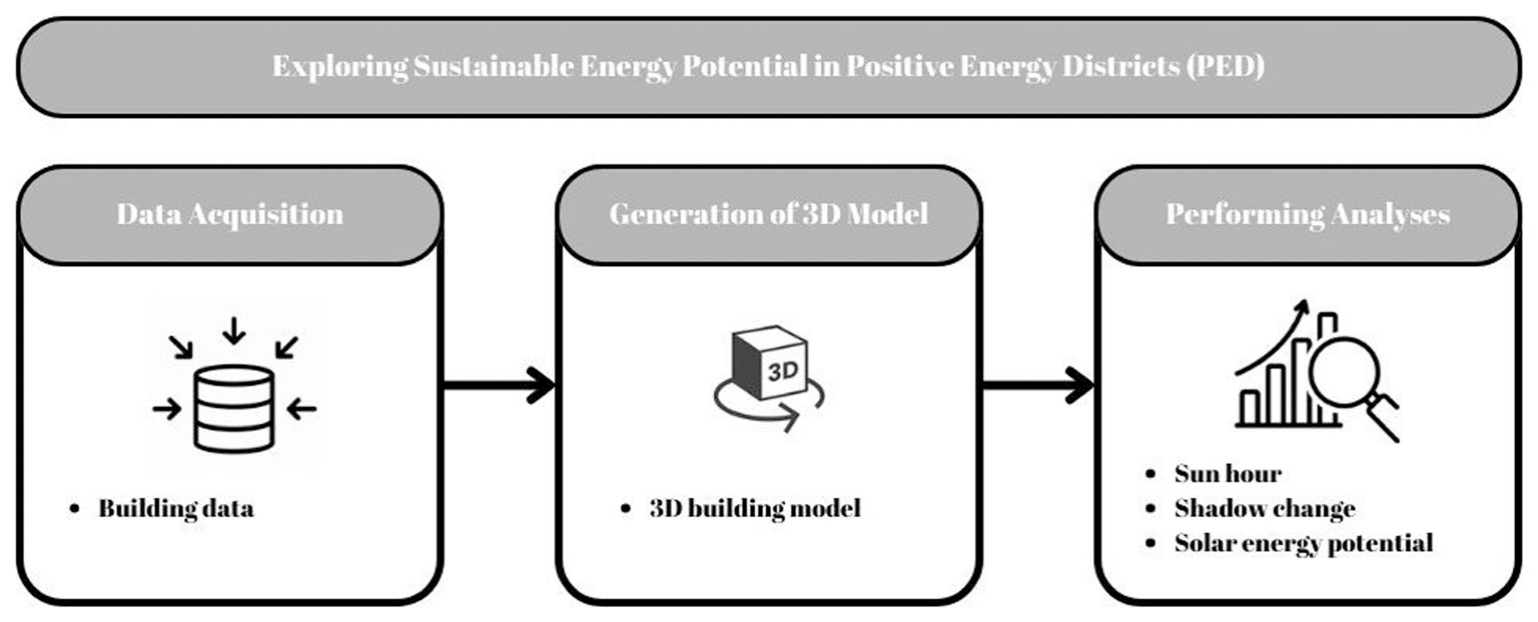
System architecture.

A GIS-based framework has been designed to integrate databases, perform spatial analyses, and support decision-making as illustrated in Figure 2. Within the scope of the study, the PED framework is supported by several spatiotemporal analyses of solar energy potentials, such as sun hour and shadow change. Since the purpose is to evaluate solar potential of different facades of buildings and roofs, three dimensional building model is essential. Two international standards are available namely, CityGML and BIM, providing high Level of Detail (LoD). However, for the study area, two-dimensional building data obtained from Open Street Map (OSM) that is freely available world-wide (Geofabrik. 2018). Download OpenStreetMap data for this region: Turkey. https://download.geofabrik.de/europe/turkey.html). Two dimensional information is transformed into three dimension using the ESRI/ArcGIS City Engine platform. (Esri. (n.d.). ArcGIS City Engine. https://www.esri.com/en-us/arcgis/products/arcgis-cityengine/overview) The height of building are taken from base-map data of ESRI for the district. The ESRI/ArcGIS City Engine platform is a proprietary software, where several alternatives exists as open source such as Blender, QGIS and CesiumJS for performing the transformation (Blender. (n.d.). Blender. https://www.blender.org/; QGIS. (n.d.). QGIS. https://www.qgis.org/; Cesium JS. (2023). Cesium JS. https://cesium.com/platform/cesiumjs/). 

The data utilized within this study are openly available in Zenodo. ( Demirel *et al.*, 2025). The spatial analyses conducted include sun hour, shadow change, and solar potential analyses performed to examine the solar energy potential of the Caferağa district. These analyses were executed using AutoDesk Forma software (Autodesk. (2024). Autodesk Forma. https://www.autodesk.com/tr/products/forma/overview?term=1-YEAR&tab=subscription). As an alternative, Python and the Pysolar and pvlib libraries and CesiumJS software can be used to exploit the solar potential (Cesium JS. (2023). Cesium JS. https://cesium.com/platform/cesiumjs/; Python. (2024). Python. https://www.python.org/; Pysolar. (n.d.). Pysolar. https://pysolar.org/; Pylib. (2022). Pylib. https://pypi.org/project/pylib-general/).

The seasonal solar potential of the buildings in the Caferağa Neighborhood was evaluated by selecting one day from each of the four seasons: winter, spring, summer, and autumn. The Sun Hour analysis on the platform examined how much the building and roof facades were exposed to the sun in different seasons. Shadow change analysis was also conducted to evaluate the time periods during the day when buildings were in the shade the most and the least. The results of the solar exposure, shadow movements, and solar potential analyses were evaluated to determine the seasonal and daily solar potential of the study area. Finally, the total and average solar potential of the buildings in the Caferağa Neighborhood were calculated based on the solar potential analysis. Obtaining detailed information about the solar potential in the region will facilitate the development of a sustainable and environmentally friendly urban infrastructure. Implementing the framework outlined here will enhance decision-making process regarding solar energy potential and facilitate the transition to a Positive Energy Districts (PED).

## Results and discussion

**Figure 3.**
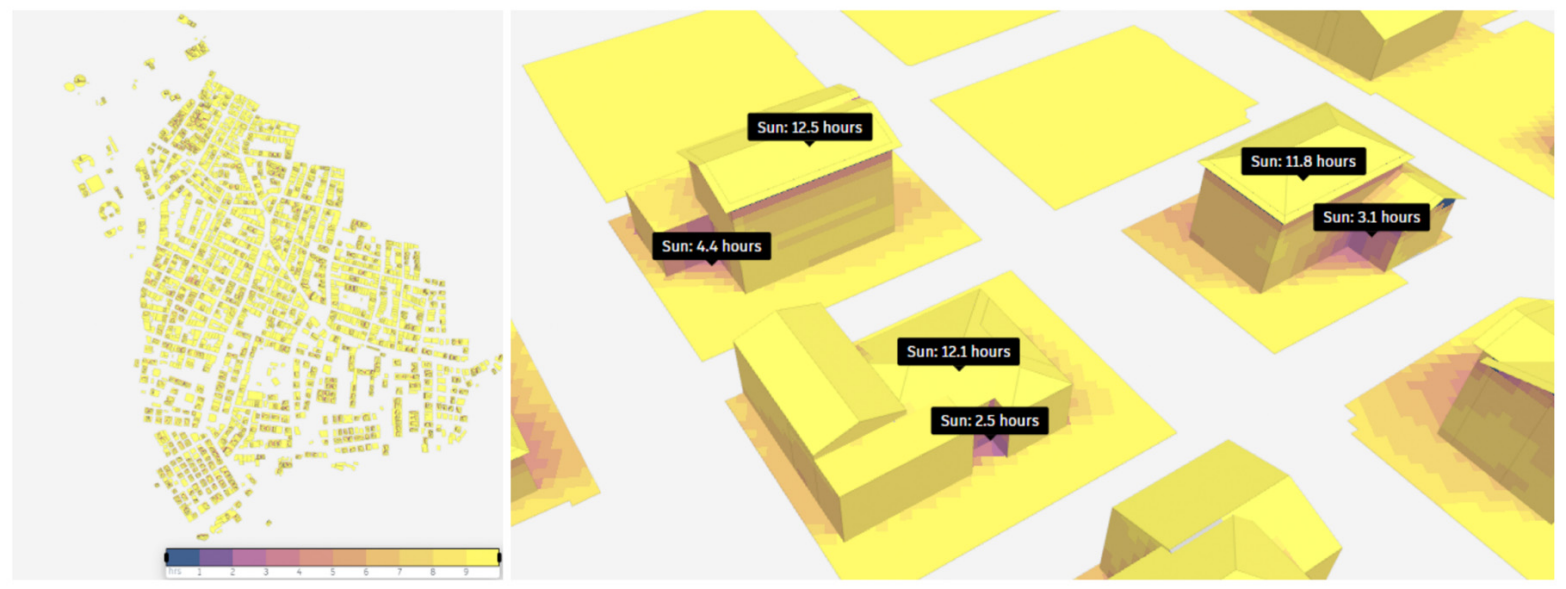
Result of sun hour analysis on May 10.

Following the framework, the 3D model of the district is achieved. The model is mainly utilized for exploring the solar energy potential, realistic visualization and effective presentation to generate a collaborative environment for all stakeholders. Furthermore, to examine the solar energy potential, the produced 3D model was used to analyze the sun hour, shadow changes, and solar energy potential. Many factors effect sunrise time, such as the angle of the sun to the earth and the region, the shadow factor, and the orientation of the building that directly effects solar energy production. Sun-hour analysis uses ray tracing technology to measure the amount of time in sunlight on a given date for points covering the ground and buildings in a specific location. The rays are tracked to determine whether the point is in the shade at a given time. For example, the solar energy potential is higher in places where there is less shade. In this study, the analysis of sun hours was conducted for May 10, August 10, October 10, and December 10. Figure 3 displays the results achieved on May 10. Additionally, this analysis does not take into consideration clouds or weather conditions, but it does account for daylight saving time, and sun rays are sampled every 6 minutes (Bakkeli, H. (2024). Introduction to the sun hours analysis. https://help.autodeskforma.com/en/articles/6951253-introduction-to-the-sun-hours-analysis).

Analysis shows that 60% of the 643.003 m ^2^ study area receives over nine hours of sunlight. The sun hours of selected buildings in the study area are also illustrated in Figure 3. This result shows that the region has a high potential for solar energy production, where maximum is reached in the spring period. According to the achieved results, it is evident that the shadows at the corners of the buildings receive minimal exposure to sunlight, while the roofs of the buildings are illuminated by the sun for almost half the day. The sun hour analysis on August 10 reveals similar pattern. In August, 60% of the study area was exposed to the sun for more than nine hours.

**Figure 4.**
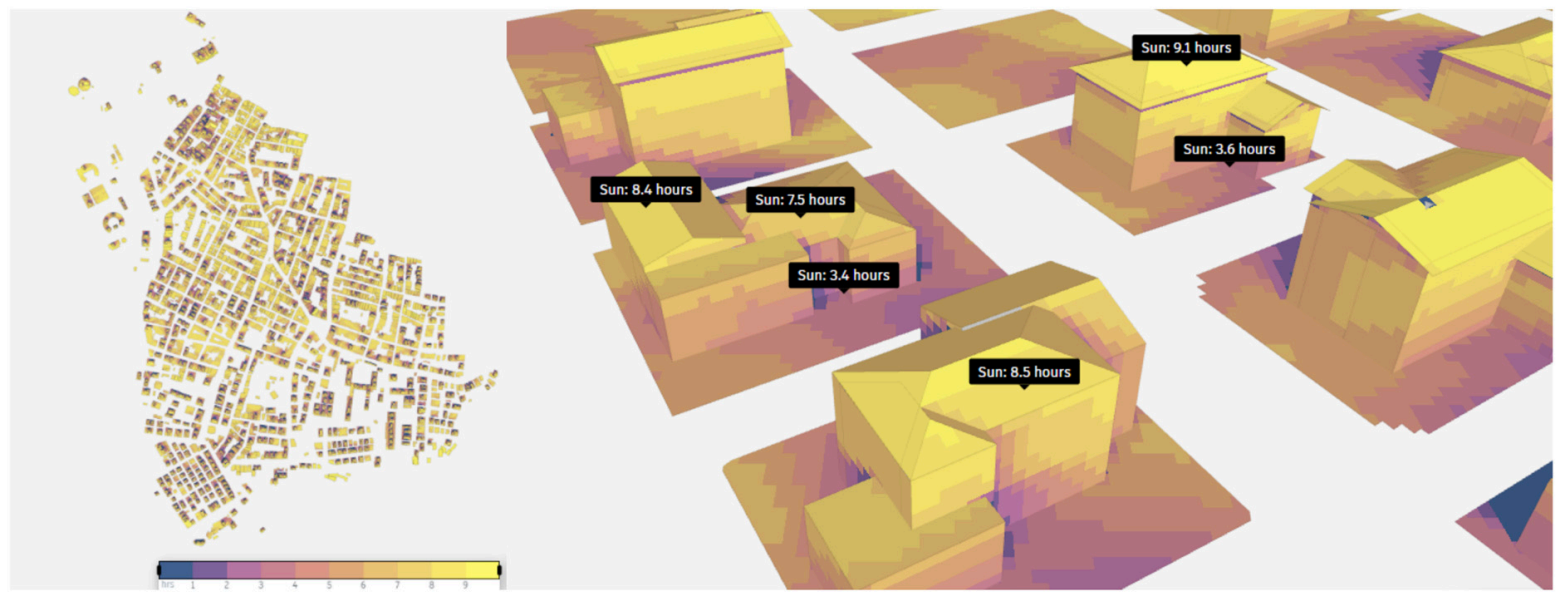
Result of sun hour analysis on December, 10th.

According to the results for the October, 10 ^th^, 49% of the study area is exposed to the sun for over nine hours. It is noted that the shadows at the corners of the buildings receive minimal sunlight, while the roofs enjoy approximately ten hours of sun exposure. According to the sun hour analysis on December, 10 ^th^, 5% of the study area is exposed to the sun for more than nine hours, and the highest area, 16%, is exposed to the sun between 7–8 hours. The sun hours of selected buildings in the study area are illustrated in Figure 4. As indicated in the figure sun exposure time for areas outside the roofs of buildings decreases, while the roofs themselves are exposed to sunlight for approximately eight hours.

**Figure 5.**
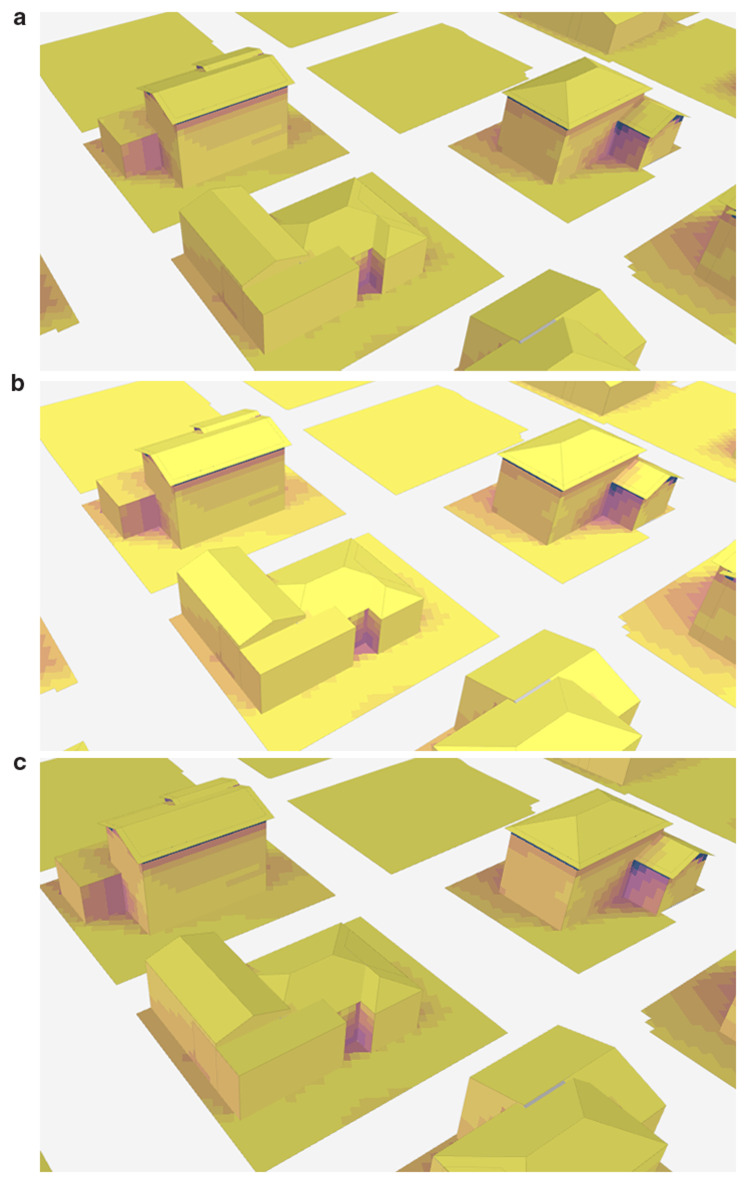
Change of shadow at different times on August 10th: (a) 7.00, (b) 14.00, and (c) 20.00.

In order to examine the shadow change during one day, the shadow conditions at 07.00 (a), 14.00 (b), and 20.00 (c) on August, 10th are examined as illustrated in Figure 5. The findings suggest that shadows are typically shorter at noon than they are in the morning and afternoon, which aligns with common expectations. During midday, buildings are more fully illuminated by the sun, leading to reduced shadow lengths. In contrast, shadows tend to be longer during sunrise and sunset, with a noticeable increase in their prominence at sunset compared to sunrise.

**Figure 6.**
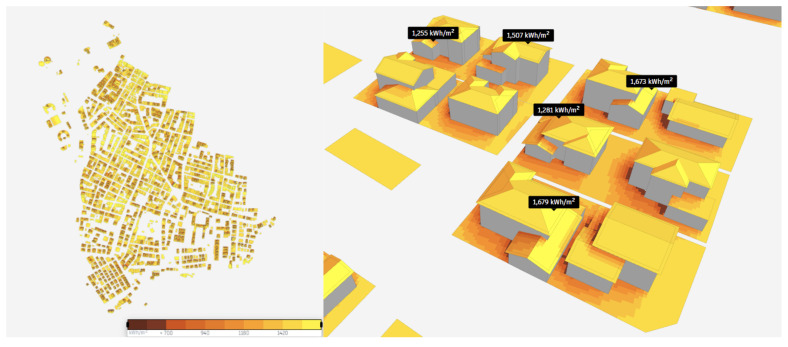
Results of the solar energy potential analysis.


In order to further explore the potential of PED, solar energy analysis is performed by extracting solar radiation data from the Copernicus database, scaling direct solar radiation for hourly sun angle assuming flat panels, and scaling diffuse solar radiation using the Isotropic sky model as illustrated in Figure 6. The solar radiation incident on a part of the surface is briefly calculated by Equation 1. In this analysis, direct solar radiation is not included for hours when a surface is in the shade, but diffuse solar radiation is included for all hours. Finally, the photovoltaic output is obtained by entering panel efficiency and roof coverage information in the analysis (Bakkeli, H. (2024). Introduction to the solar energy analysis. https://help.autodeskforma.com/en/articles/8281900). In this study, efficiency is taken as 15% based on the fact that the efficiency of most solar panels is between 15% and 20% (Vourvoulias, A. (2014,). How efficient are solar panels? https://www.greenmatch.co.uk/blog/2014/11/how-efficient-are-solar-panels). Finally, in the study, the surface coverage parameter, which expresses the percentage of surfaces covered by productive solar panels excluding the supporting infrastructure, was taken as 60%, and analyses were conducted.

TotalIncidentRadiation=(SunResults∗DirectSolarRadiation)+DiffuseSolarRadiation(1)

According to the results of the analysis, the annual solar energy potential of the study region was reached. The total solar energy was 629,000,000 kWh, and the average solar energy was 1,410 kWh/m2. The solar energy potentials of sample buildings in the study region are shown in Figure 6. It is observed that the solar energy potential is high on the roofs and different facades of the building as well.

## Conclusion

In this study, a spatial framework was developed to evaluate the sustainable energy potential of Positive Energy Districts (PEDs). The developed concepts have been thoughtfully implemented in the Caferağa Neighborhood of Kadiköy, İstanbul, Turkey. A range of spatial analyses has been conducted, encompassing solar energy assessments, sunlight exposure evaluations, and the examination of shadow changes throughout the day. To account for seasonal variations, analyses were performed on one specific day from each of the four seasons: May 10, August 10, October 10, and December 10. The results of the insolation analysis revealed that buildings in the area received sunlight for nearly half the day during the spring and summer months. However, this duration decreased in the fall and significantly declined during the winter.

With the current technology and state-of-art, renewable energy sources such as solar energy can be harnessed efficiently despite the region's limited sunlight availability. Analyzing the study area in this context reveals that solar energy production remains viable during the fall and winter seasons, even though sun exposure is less compared to other times of the year. Another factor influencing insolation time is location. It was noted that corner points situated more towards the interior of the building are generally shaded, resulting in minimal sun exposure. When the shadow change during the day was examined on August 10, when the sun hour duration was the highest, it was observed that the shadow was less at noon and the shadow was more at sunrise and sunset. Since there is more exposure to the sun when the shadow is high, the sun exposure time is higher at noon when the shadow is low. Finally, when the solar energy potential of the study area is analyzed, it is observed that the solar energy potential is high, especially on the roofs of the buildings. However, the solar energy potential on the roofs also differs between the facades. At the end of the study, it was concluded that the total solar energy in the study region is 629,000,000 kWh, and the average solar energy is 1,410 kWh/m2. However, since the parameters that may affect the feasibility such as panel type and number of panels were not included in the analysis, it is foreseen that the results can be improved by including these parameters in future studies.

Since the entire potential roof area can be utilized, the primary energy is higher for PV panels in detached and small houses. When the building structure in the study region is analyzed, the fact that there are no very high-rise buildings in the region supports the results obtained and shows that the region is suitable for solar energy use. However, parameters such as the year of construction, materials used, and building durability should also be evaluated in order to make precise inferences. The estimated annual electricity production of PV panels is 212.11 GWh/y, but this value becomes 211.5 GWh/y when the maximum potential roof area for solar thermal energy production is taken into account ( Horváth *et al.*, 2016). As a result of the analysis carried out within the scope of the study, it was observed that the annual solar energy potential of the study region with a production of 629 GWh is 33.62% higher than the values within the literature.

This study discusses the factors influencing energy production from renewable sources, particularly solar energy, and explores methods for their analysis. To evaluate solar energy potential, we examined various building facades and roofs to determine the number of hours they receive sunlight each day and identify areas that are more shaded due to changing shadows throughout the day. Additionally, these parameters were assessed seasonally, revealing that buildings receive the most sunlight during the summer, which positively impacts solar energy production. Another aspect of the study addresses the type of data required for these analyses and how the quality of this data affects the results. Evaluating different building facades necessitates 3D data. In this study, we addressed this challenge by generating a 3D model using available two dimensional (2D) building data, including floor information, and conducting analyses on this model. However, for a comprehensive evaluation of the various parameters affecting solar energy potential, we recommend using BIM, which contains essential information about the building and its materials. Furthermore, acquiring more detailed atmospheric and regional 2D and 3D information will facilitate a more accurate and robust assessment of solar energy potential.

The analysis indicates that designating the study area as a PED would allow for significant solar energy generation from most buildings, particularly during the spring and summer months. This potential suggests that a PED can effectively produce sustainable energy and contribute to fulfilling the area’s energy requirements. However, to conclusively assess the area's suitability as a PED, it is essential to evaluate various additional factors such as energy grids, business models and economic factors. Additional key considerations include the number and type of solar panels utilized, the energy potential of alternative renewable energy sources, and additional parameters that influence PED efficiency, such as transportation infrastructure, social awareness, and the arrangement of buildings within the district.

## Ethics and consent

Ethical approval and consent were not required.

## Data Availability

Zenodo: Exploring the sustainable energy Potential of Positive Energy Districts (PED) via Geographic Information System (GIS), https://doi.org/10.5281/zenodo.14604612 [ Demirel *et al.*, 2025] *This projects contains the following underlying data:* - *[buildings_caferaga_osm.cpg] (The provided data includes information about buildings in the Caferağa district of Kadikoy, Istanbul- Türkiye. All components of the sample dataset needs to be downloaded to function correctly.)* - *[buildings_caferaga_osm.dbf] (The provided data includes information about buildings in the Caferağa district of Kadikoy, Istanbul- Türkiye. All components of the sample dataset needs to be downloaded to function correctly.)* - *[buildings_caferaga_osm.prj] (The provided data includes information about buildings in the Caferağa district of Kadikoy, Istanbul- Türkiye. All components of the sample dataset needs to be downloaded to function correctly.)* - *[buildings_caferaga_osm.qmd] (The provided data includes information about buildings in the Caferağa district of Kadikoy, Istanbul- Türkiye. All components of the sample dataset needs to be downloaded to function correctly.)* - *[buildings_caferaga_osm.shp] (The provided data includes information about buildings in the Caferağa district of Kadikoy, Istanbul- Türkiye. All components of the sample dataset needs to be downloaded to function correctly.)* - *[buildings_caferaga_osm.shx] (The provided data includes information about buildings in the Caferağa district of Kadikoy, Istanbul- Türkiye. All components of the sample dataset needs to be downloaded to function correctly.)* - *[buildings_caferaga_osm.csv] (The provided data includes information about buildings in the Caferağa district of Kadikoy, Istanbul- Türkiye.)* *The study utilizes open-source data from Open Street Map (OSM).* Data is available under Creative Commons Attribution 4.0 International (CC BY 4.0 license).

## Software availability statement

The ESRI/ArcGIS City Engine platform is a proprietary software, where several alternatives exists as open source such as Blender, QGIS and CesiumJS for performing the transformation (Esri. (n.d.). ArcGIS City Engine. https://www.esri.com/en-us/arcgis/products/arcgis-cityengine/overview; Blender. (n.d.). Blender. https://www.blender.org/; QGIS. (n.d.). QGIS. https://www.qgis.org/; Cesium JS. (2023). Cesium JS. https://cesium.com/platform/cesiumjs/).

The AutoDesk Forma software is a proprietary software. As an alternative, Python and the Pysolar and pvlib libraries and CesiumJS software can be used to exploit the solar potential (Cesium JS. (2023).

Cesium JS. https://cesium.com/platform/cesiumjs/;

Autodesk. (2024). Autodesk Forma. https://www.autodesk.com/tr/products/forma/overview?term=1-YEAR&tab=subscription;

Python. (2024). Python. https://www.python.org/;

Pysolar. (n.d.). Pysolar. https://pysolar.org/;

Pylib. (2022). Pylib. https://pypi.org/project/pylib-general/).
