# Evaluating the solar energy potential for Positive Energy Districts (PED) through advanced 3D Geographic Information System (GIS) analysis

**DOI:** 10.12688/openreseurope.19075.2

**Published:** 2025-07-14

**Authors:** Hande Demirel, Ayşenur Koçyiğit, Ben Alpagut, Damla Muhcu

**Affiliations:** 1 Faculty of Civil Engineering, Department of Geomatics Engineering, Istanbul Technical University, İstanbul, 34469, Turkey; 2 Faculty of Civil Engineering, Department of Geomatics Engineering, Istanbul Technical University, İstanbul, 34469, Turkey; 3 Smart and Sustainable Cities Department, Demir Energy, İstanbul, 34718, Turkey; 4 Department of Project Management, Municipality of Kadıköy, İstanbul, 34716, Turkey

**Keywords:** Positive Energy District (PED), Renewable Energy, Geographic Information Systems (GIS), Solar Energy, Smart Cities, Sustainability

## Abstract

**Background:**

The Positive Energy District (PED) concept aims to transform urban areas into carbon-neutral and zero-energy communities by reducing energy consumption and increasing renewable energy production. However, challenges remain in making effective decisions, including inconsistent data sources, difficulties in collaboration among stakeholders, and the need to merge interdisciplinary technical and scientific knowledge. Hence, GIS-based frameworks offer mature solutions to address such issues.

**Methods:**

This study introduces a three-dimensional (3D) GIS-based framework designed to improve decision-making in PEDs, with a particular focus on spatiotemporal analyses of solar energy potential. The rationale behind selecting solar energy is the high potential of urban areas for suitability, as well as the legal and technical constraints associated with wind and hydro energy production within densely populated areas and built-up areas. The literature review showed that the combined use of 3D GIS and PED concepts has not yet been widely explored, despite the significant potential that exists. The developed framework were deployed and tested in the case study area – the Caferağa neighborhood of Kadıköy, Türkiye. In order to test and validate the achieved results, various platforms are utilized and compared.

**Results:**

The 3D GIS-based framework demonstrated several advantages over traditional systems. It utilized spatial data, including high-resolution 3D building models, to assess solar potential, employing and comparing various spatial tools. Comprehensive analyses were performed to assess the exposure of buildings to sunlight during different seasons and to evaluate the shading effects encountered throughout the day. The analysis of sun hour revealed that only 5% of the entire work area received more than nine hours of sunlight during the winter. Additionally, when evaluating the solar potential, a 22.29% discrepancy was found within utilized platforms.

**Conclusion:**

The achieved results are promising, where co-creation, co-design- and co-implementation could be performed collaboratively via designed platform that seamlessly integrate data, functions, necessary tools and expertise. The results indicate that 3D spatial analyses promote the potential for solar energy and aid decision-makers in the complex processes of designing and expanding PEDs.

## Introduction

The primary goal of the Positive Energy District (PED) is to generate more energy than the district consumes, thus creating a surplus through renewable technologies. In essence, PEDs strive to establish sustainable communities that rely entirely on renewable energy sources. However, it is a challenge for several districts worldwide, since it is vital for new buildings constructed in the area to meet energy efficiency targets, where, existing buildings must be transformed and improved to enhance their energy efficiency ( Jepsen *et al.*, 2022). Furthermore, each PED must establish an optimal balance among energy efficiency, energy flexibility, and local energy production to achieve climate neutrality and an energy surplus (Urban Europe. (2020). Reference Framework for Positive Energy Districts and Neighbourhoods. https://jpi-urbaneurope.eu/wp-content/uploads/2020/04/White-Paper-PED-Framework-Definition-2020323-final.pdf). The Positive Energy District (PED) concept highlights the interaction among energy production, consumers, and storage within a region ( Lindholm *et al.*, 2021). Developed collaboratively, PEDs aim to foster sustainable urban development and transition cities to climate-neutral energy systems ( Turci *et al.*, 2022). The energy-efficient solutions require more data-driven management and control strategies, since such concepts must effectively address the needs of its users while reducing energy consumption and environmental impact. ( Ala-Juusela *et al.*, 2021).

To respond such challenges, several research projects are recently accomplished, where a comprehensive list is provided below in Table 1. (ATELIER. (2024). AmsTErdam BiLbao cItizen drivEn smaRt cities. https://smartcity-atelier.eu/; CityxChange. (n.d.). CityxChange. https://cityxchange.eu/; MAKING-CITY. (2019). Making City. https://makingcity.eu/; POCITYF. (n.d.). POCITYF. https://pocityf.eu/; Stardust. (n.d.). STARDUST: Enlightening European Cities. https://stardustproject.eu/).

**Table 1.** Horizon 2020 PED Projects. ATELIER. (2024). AmsTErdam BiLbao cItizen drivEn smaRt cities. https://smartcity-atelier.eu/; CityxChange. (n.d.). CityxChange. https://cityxchange.eu; MAKING-CITY. (2019). Making City. https://makingcity.eu/; POCITYF. (n.d.). POCITYF. https://pocityf.eu/; Stardust. (n.d.). STARDUST: Enlightening European Cities. https://stardustproject.eu/).

Name	Scope	Date
MAKING-CITY	The aim of this project is to demonstrate the transformation of urban energy systems towards smart, low-carbon cities using the concept of PED.	2018–2024
STARDUST	STARDUST is a smart cities project focused on transforming cities that emit carbon into highly efficient, smart, and citizen-oriented urban environments. The project aims to develop innovative green solutions and business models while integrating building design, mobility, and energy efficiency.	2017–2024
CityxChange	The goal is to develop a framework and supporting tools to enable a unified energy market, foster a connected community, and provide recommendations for new policy interventions, market liberalization, and business models. These initiatives aim to support positive energy communities and integrate e-Mobility as a Service (eMaaS).	2018–2023
POCITYF	POCITYF is a smart city project designed to help historical cities become greener, smarter, and more livable while preserving their cultural heritage.	2019–2024
ATELIER	The project aims to enhance the local innovation ecosystem by conducting PED Innovation Workshops, which will help eliminate legal, financial, and social barriers to implementing smart solutions. These workshops will become self-sustaining after the project concludes, serving as engines for scaling and replicating solutions in ATELIER cities and beyond.	2019–2024

These projects address various aspects of PED challenges. MAKING-CITY aims to enhance citizen engagement by developing robust city visions and facilitating knowledge sharing to create district energy plans and PEDs through a bottom-up approach. The MAKING-CITY project is benefits from Geographic Information System (GIS)-based spatial analyses. STARDUST has tested technical green solutions and non-technical approaches, demonstrating their bankability and replicability. It offers valuable insights for cities pursuing climate neutrality and sustainable development (STARDUST: Enlightening European Cities. https://stardustproject.eu/). CityxChange has developed a toolbox for managing local energy systems, helping districts become Positive Energy Blocks (PEBs). It provides grid topology information, estimates generation and load, and evaluates the impact of local resources ( Dahlen *et al.*, 2020). POCITYF unites technology providers, local governments, and citizens to create integrated and innovative solutions that facilitate the energy transition and create PEDs. Within this framework, various mechanisms are being piloted and tested in selected regions, including peer-to-peer (P2P) energy trading, token systems that reward sustainable behavior, and digital platforms designed to enhance citizen engagement (POCITYF. (n.d.). POCITYF. https://pocityf.eu/). ATELIER focuses on establishing smart grids within a community-owned framework, enabling the sharing, distribution, and trading of renewable energy among individuals and organizations. These projects integrate physical investments with social engagement in the energy transition, promoting collaboration among stakeholders. They develop multi-level strategies and support energy citizenship, showcasing the benefits of unified planning sought by many European cities. ( Olivadese *et al.*, 2021).

Another recent important program funded by JPI Urban Europe, namely Positive Energy Regions and Cities (ENPED), is designed to enhance the research and innovation capacity of the European Union (EU) in pursuit of climate neutrality across 100 regions by 2030. One of the projects funded under this program being the Positive Robust PED Localities (PROPEL), where authors are also contributing, aims to include innovative PED components and improving possibilities for off-grid operation to increase the robustness and viability of PEDs, utilizing viable business models and innovative types of energy carriers via recycling of energy in the food-feed system, biogas and including transports in the PED system of systems. Within these projects, mainly GIS-based decision support systems are designed and deployed to ensure the collaborative environment that is essential.

Several national PED projects benefit from spatial analyses to test cost-effectiveness of PEDs. For example, according to a study conducted in the Alfama district in Lisbon, the potential reduction in annual energy demand with building rehabilitation was 84% for space heating and 19% for cooling. ( Gouveia *et al.*, 2021). At the same time, the integration of building-integrated Photovoltaics (PV) technologies on roofs and windows potentially provides production up to 60 GWh/year. When evaluated on a regional scale, these two components of the PED concept can require an investment of 60M€ to 81M€, depending on the PV technologies used on roofs, which is a challenging aspect in historic districts ( Gouveia *et al.*, 2021).

The PED concept has a very strong spatial pillar, where GIS is a mature ecosystem for successfully managing spatial data-driven challenges. Especially, decision-making for planning, managing and operating such systems requires support from spatial analyses and multi-criteria decision-making. Furthermore, via such ecosystems, visualization of PEDs to various stakeholders, including citizens, could be successfully performed ( Alpagut *et al.*, 2021). Hence, there are several recent studies that integrate PED and GIS successfully ( Alpagut *et al.*, 2019; Lindholm *et al.*, 2021; Zhang *et al.*, 2021a), where in Table 2, some of them are shown. While these studies provide valuable insights, they do have some limitations, including their two-dimensional nature, lower spatial resolution, and certain data quality issues that may affect their potential for integration.

**Table 2.** Research in the literature that focuses on spatial analysis and PED.

Resource	Reference	Spatial Analyses	PED
Positive Energy Districts Methodology and Its Replication Potential	Alpagut *et al.*, 2019	✔	✔
Multi-criteria decision making for solar power - Wind power plant site selection using a GIS-intuitionistic fuzzy-based approach with an application in the Netherlands	Şahin *et al.*, 2024	✔	**X**
A GIS-Based Multicriteria Assessment for Identification of Positive Energy Districts Boundary in Cities	Alpagut *et al.*, 2021	✔	✔
Positive Energy District: A Model for Historic Districts to Address Energy Poverty	Gouveia *et al.*, 2021	✔	✔
GIS-based solar radiation modelling for photovoltaic potential in cities: A sensitivity analysis for the evaluation of output variability range	Anselmo *et al.*, 2024	✔	**X**
GIS-Based Digital Twin Model for Solar Radiation Mapping to Support Sustainable Urban Agriculture Design	Clementi *et al.*, 2024	✔	**X**
Enhancing rooftop solar energy potential evaluation in high-density cities: A Deep Learning and GIS based approach	Ni *et al.*, 2023	✔	**X**
GIS-based assessment of photovoltaic solar potential on building rooftops in equatorial urban areas	Idrovo-Macancela *et al.*, 2024	✔	**X**
Pathways to urban net zero energy buildings in Canada: A comprehensive GIS- based framework using open data	Li & Feng, 2025	✔	**X**
Multicriteria Spatial Economic Decision Support Systems to Support Positive Energy Districts: A Literature Review	Bisello *et al.*, 2023	✔	✔
Digital Twin for Accelerating Sustainability in Positive Energy District: A Review of Simulation Tools and Applications	Zhang *et al.*, 2021b	✔	✔

In conjunction with the studies outlined in Table 2, there are several promising initiatives that thoughtfully explore the application of Digital Twins (DTs) within the framework of PED concepts. Digital twins are three-dimensional models enhanced by sensors that replicate the physical world. They are typically used for monitoring and scenario-based decision-making. Notably, the research conducted by Shen *et al.* (2021), Coors and Padsala (2024), and Malakhatka *et al.* (2024) has thoughtfully integrated digital twins with the concept of PED, highlighting the potential for innovative applications in this field. In addition to these studies, there are ongoing projects that incorporate DT and PED concepts, one of which is the European Union-supported DigitalTwin4PEDs project (DigitalTwin4PEDs. (2025). DigitalTwin4PEDs. https://digitwins4peds.eu/) Furthermore, Shen *et al.* (2021), Coors & Padsala (2024), and the DigitalTwin4PEDs project utilized GIS-based DTs, while Malakhatka *et al.* (2024) employed another three-dimensional model, Building Information Modelling (BIM), for digital twins. Hence, the core idea of using Digital Twins (DT) with PED systems is based on three-dimensional spatial information. Through effective 3D Geographic Information System (GIS) analyses, we can enhance the decision-making process, leading to more informed and effective outcomes.

After carefully analyzing Table 2, it is evident that there is a significant opportunity for further research that combines PED with spatial analyses, particularly about the potential of solar energy. This is particularly relevant in complex urban areas, where the adoption of various renewable energy resources encounters multiple constraints. Additionally, the use of three-dimensional (3D) GIS has not been investigated in the context of PED, and adopting this approach could reveal valuable insights and new research opportunities.

This study aims to propose a comprehensive approach that deploys 3D - GIS to explore the solar energy potential for PED. This study focuses on the research questions outlined below.

-   What are the factors influencing energy production from renewable sources, particularly solar energy? How can this be analyzed?

-   What types of data–both spatial and non-spatial–are necessary for conducting such analyses? What are the potential benefits of using 3D data?

The concepts are being tested in the PED district of Kadıköy, Türkiye, where the complex urban structure presents additional challenges that could also be addressed using GIS. In order to promote replicability and facilitate broader implementation of the designed framework, open-source data, and software platforms were utilized.

## Methods

**Figure 1.**
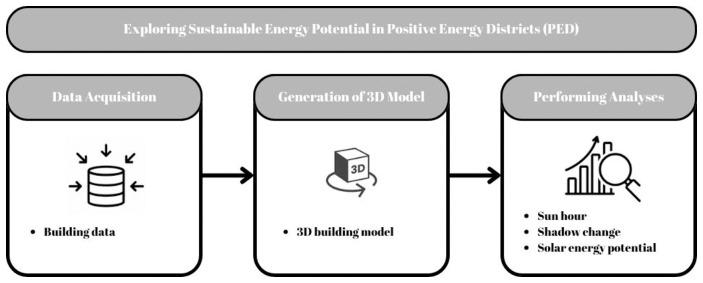
System architecture.

A GIS-based framework has been designed to integrate databases, perform spatial analyses, and support decision-making, as illustrated in Figure 1. Within the scope of the study, the PED framework is supported by several spatiotemporal analyses to determine solar energy potential, including sun hours and shadow changes. Since the purpose is to evaluate the solar potential of different facades of buildings and roofs, 3D building model is essential. In this study, a 3D model was developed using two-dimensional (2D) building data obtained from OpenStreetMap (OSM). OSM was chosen as the data source because it is an open-source platform, which enhances the replicability of the study on a global scale. Additionally, analyses can also be performed using other 3D data models such as City Geography Markup Language (CityGML) and BIM. https://download.geofabrik.de/europe/turkey.html). 2D information was converted into three dimensions using both the ESRI/ArcGIS CityEngine platform and Blender, as the building roofs could only be fully represented in CityEngine. (Esri. (n.d.). ArcGIS City Engine. https://www.esri.com/en-us/arcgis/products/arcgis-cityengine/overview; Blender. (n.d.). Blender. https://www.blender.org/) The height information of buildings were taken from base-map data of ESRI for the district. The ESRI/ArcGIS City Engine platform is a proprietary software, where several alternatives exists as open source such as QGIS and CesiumJS for performing the transformation (Blender. (n.d.). Blender. https://www.blender.org/; QGIS. (n.d.). QGIS. https://www.qgis.org/; Cesium JS. (2023). Cesium JS. https://cesium.com/platform/cesiumjs/).

The data utilized within this study are openly available in Zenodo. ( Demirel *et al.*, 2025). The spatial analyses conducted include sun hour, shadow change, and solar potential analyses. The analysis was conducted using three distinct tools: AutoDesk Forma, PVGIS, and Ladybug Tools. In particular, PVGIS and Ladybug Tools were thoughtfully selected to carry out sensitivity analyses and validate the results, ensuring a comprehensive understanding of the findings. (Autodesk. (2024). Autodesk Forma. https://www.autodesk.com/tr/products/forma/overview?term=1-YEAR&tab=subscription; Photovoltaic Geographical Information System. (2024). PVGIS. https://re.jrc.ec.europa.eu/pvg_tools/en/; Ladybug Tools. (2022). Ladybug Tools. https://www.ladybug.tools/). As an alternative to AutoDesk Forma, Python and the Pysolar and pvlib libraries and CesiumJS software can be used to exploit the solar potential (Cesium JS. (2023). Cesium JS. https://cesium.com/platform/cesiumjs/; Python. (2024). Python. https://www.python.org/; Pysolar. (n.d.). Pysolar. https://pysolar.org/; Pylib. (2022). Pylib. https://pypi.org/project/pylib-general/). PVGIS, developed by the European Commission, is an open-source platform that allows for the assessment of solar radiation and photovoltaic (PV) system performance on a global scale, excluding the polar regions. The analysis utilizes SARAH-2 solar radiation data, which is derived from METEOSAT satellite imagery, providing long-term hourly averages from 2005 to 2020. (Photovoltaic Geographical Information System. (2024). PVGIS. https://re.jrc.ec.europa.eu/pvg_tools/en/). Ladybug Tools facilitates the visualization and analysis of meteorological information within the Grasshopper environment. For the Direct Sun Hours analysis, The LB SunPath and EPW Map components were used to integrate solar vectors with local meteorological data, allowing for an assessment of seasonal solar exposure (Ladybug Tools. (2022). Ladybug Tools. https://www.ladybug.tools/; Grasshopper. (2025). Ladybug Tools. https://www.grasshopper3d.com/group/ladybug; Rhino (2025). Ladybug Tools. https://www.food4rhino.com/en/app/ladybug-tools; Ladybug Primer. (2024). Direct Sun Hours. https://docs.ladybug.tools/ladybug-primer/components/3_analyzegeometry/direct_sun_hours).

**Figure 2.**
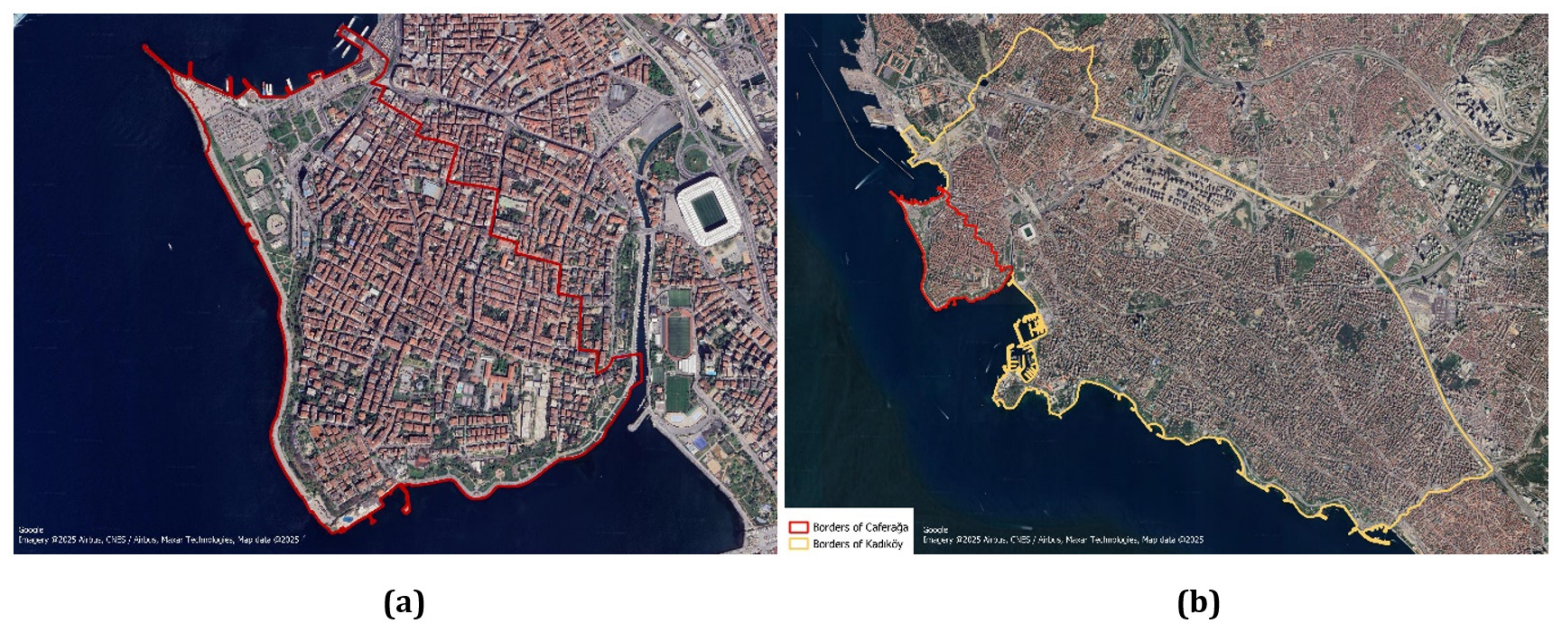
( **a**) Borders of Caferağa, ( **b**) Caferağa neighborhood in Kadıköy district (Google. (2022). Google Maps/Google Earth Additional Terms of Service. https://www.google.com/intl/en_tr/help/terms_maps/)

The developed concepts are tested and validated in a case study area. While the core approach and methodology have universal significance, it is important to tailor the PED to fit the local context. This study takes place in the Caferağa district of Kadiköy, Istanbul, Türkiye, where various noteworthy PED and sustainability projects and initiatives are currently being implemented. Istanbul, located in the Marmara Region of Turkey, is the country's most populous city, boasting a population of 15,907,951 (Turkish Statistical Institute (TURKSTAT). (2022). Adrese Dayalı Nüfus Kayıt Sistemi Sonuçları. https://data.tuik.gov.tr/Bulten/Index?p=49685#:~:text=%C4%B0stanbul'un%20n%C3%BCfusu%2015%20milyon,907%20bin%20951%20ki%C5%9Fi%20oldu). Spanning an area of 5,712 km², Istanbul serves as a strategically significant metropolitan area that bridges the continents of Europe and Asia continents. Its prominence as a major trade center contributes to its high population and traffic density. The Kadiköy Municipality has been selected as the pilot area for the MAKING-CITY and PROPEL projects, highlighting Türkiye's participation in the PED initiative. This selection emphasizes the district's capacity to lead innovative urban development efforts. Furthermore, Sustainable Urban Mobility Plan (SUMP) developed by the Istanbul Metropolitan Municipality (IBB) designates the Caferağa Neighborhood as a low-emission zone. Caferağa Neighborhood is one of the oldest neighborhoods in Kadıköy. The total area of the neighborhood is 1,2374 km ^2^, of which 0.028 km² are residential areas, as illustrated in Figure 2. The economic viability of the neighborhood is quite high, with a Socio-Economic Status (SES) score of 100 in 2016 (Istanbul Metropolitan Municipality, Urban Planning Department. (n.d.). Caferağa Mahallesi. https://sehirplanlama.ibb.istanbul/caferaga-mahallesi/; The Istanbul Electricity, Tram and Tunnel. (IETT) (2024). IETT GTFS Data. https://data.ibb.gov.tr/en/dataset/iett-gtfs-verisi; Municipality of Kadiköy. (n.d.). Anlat Kadıköy. https://anlat.kadikoy.bel.tr/mahalleler/caferaga).

According to the designed frame-work, the seasonal solar potential of the buildings in the Caferağa Neighborhood was evaluated by selecting one day from each of the four seasons: winter, spring, summer, and autumn. The Sun Hour analysis on the platform examined how much the building and roof facades were exposed to the sun in different seasons. Shadow change analysis was also conducted to evaluate the periods during the day when buildings were in the shade the most and the least. The results of the solar exposure, shadow movements, and solar potential analyses were evaluated to determine the seasonal and daily solar potential of the study area. Finally, the total and average solar potential of the buildings in the Caferağa Neighborhood were calculated based on the solar potential analysis. Obtaining detailed information about the solar potential in the region will facilitate the development of a sustainable and environmentally friendly urban infrastructure. Implementing the framework outlined here will enhance decision-making process regarding solar energy potential and facilitate the transition to a PED.

## Results and discussion

In accordance with the established framework, a high-resolution 3D model of the district has been successfully developed utilizing open-source platforms. The model could be utilized for exploring the solar energy potential, providing realistic visualization, and effective presentation to generate a collaborative environment for all stakeholders. Furthermore, to examine the solar energy potential, the produced 3D model was used to analyze the sun hour, shadow changes, and solar energy potential.

**Figure 3.**
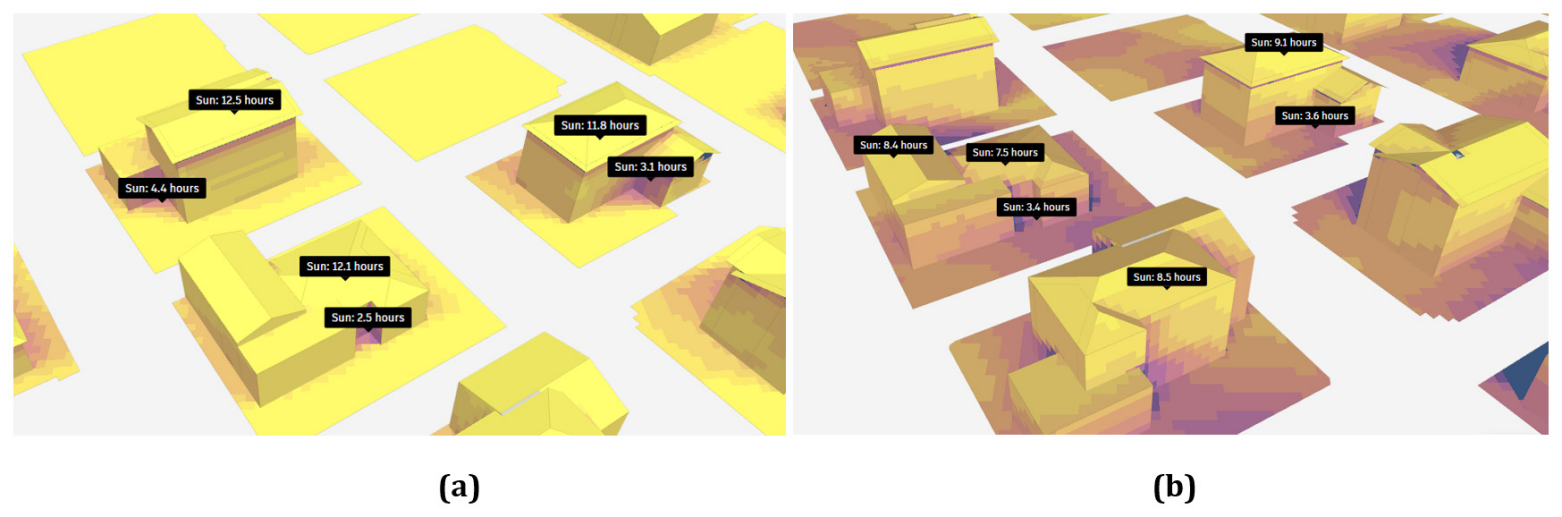
Result of sun hour analyses on ( **a**) May 10 and ( **b**) December 10.

In this study, the analysis of sun hours was conducted for May 10, August 10, October 10, and December 10. Figure 3 displays the results achieved on May 10 and December 10. Additionally, this analysis does not take into consideration clouds or weather conditions, but it does account for daylight saving time, and sun rays are sampled every 6 minutes (Bakkeli, H. (2024). Introduction to the sun hours analysis. https://help.autodeskforma.com/en/articles/6951253-introduction-to-the-sun-hours-analysis).

Analyses show that 60% of the 643,003 m² working area receives more than nine hours of sunlight. The results of the solar hour analysis for the selected buildings in the study area on May 10 are presented in Figure 3a, and the results for December 10 are shown in Figure 3b. This result indicates that the region has a high potential for solar energy production, where the maximum was reached in the spring period. According to the achieved results, it is evident that the shadows at the corners of the buildings receive minimal exposure to sunlight, while the roofs of the buildings are illuminated by the sun for almost half the day. The sun hour analysis conducted on August 10th was similar to the analysis performed on May 10th.

The analysis conducted for August reveals that 60% of the work area is exposed to sunlight for more than nine hours. In October, this percentage is observed to drop to 49% and further declines to 5% in December. Notably, when assessing the buildings, it was found that in October, the corners received limited sunlight, whereas the roofs enjoyed exposure for approximately ten hours. In December, as depicted in Figure 3, the duration of sunlight exposure for areas other than the roofs decreased, with the roofs receiving around eight hours of sunlight.

**Figure 4.**
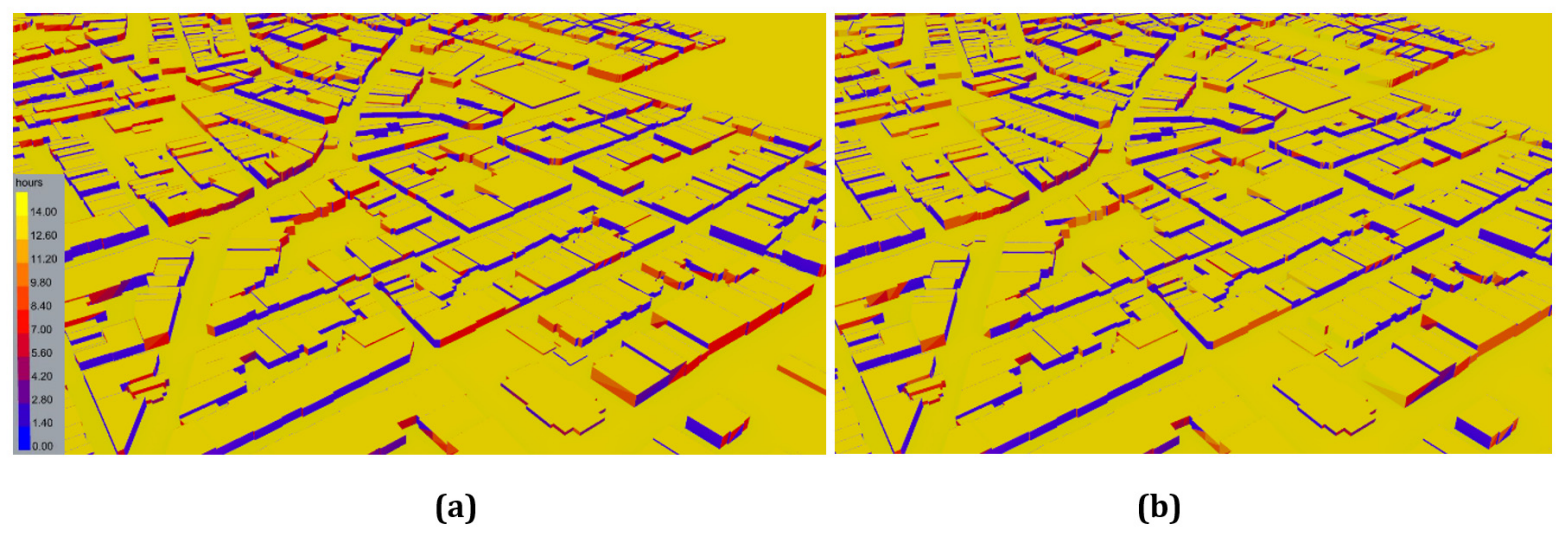
Direct Sun Hours Analysis Results of ( **a**) August 10 and ( **b**) December 10.

In order to validate the results of the sun hour analyses, the Ladybug tools were used, where the results are illustrated in Figure 4. The analysis results for August 10, as shown in Figure 4a, indicate that the south and southeast facades of buildings are most exposed to sunlight. On this date, the maximum sun exposure time reached 14 hours. Additionally, the results indicate that the duration of sun exposure increases with the number of floors in the buildings. The analysis results for December 10, as shown in Figure 4b, reveal that the maximum duration of sun exposure in December is 9 hours. Furthermore, it was noted that the duration of exposure to sunlight decreased by up to 35.7% when comparing winter and summer. The findings from Autodesk Forma show that only 5% of the entire study area receives direct sunlight for more than nine hours. In contrast, the results obtained using the Ladybug methodology do not specify the percentage of the study area exposed to direct sunlight; only the maximum duration of exposure is indicated as nine hours.

**Figure 5.**
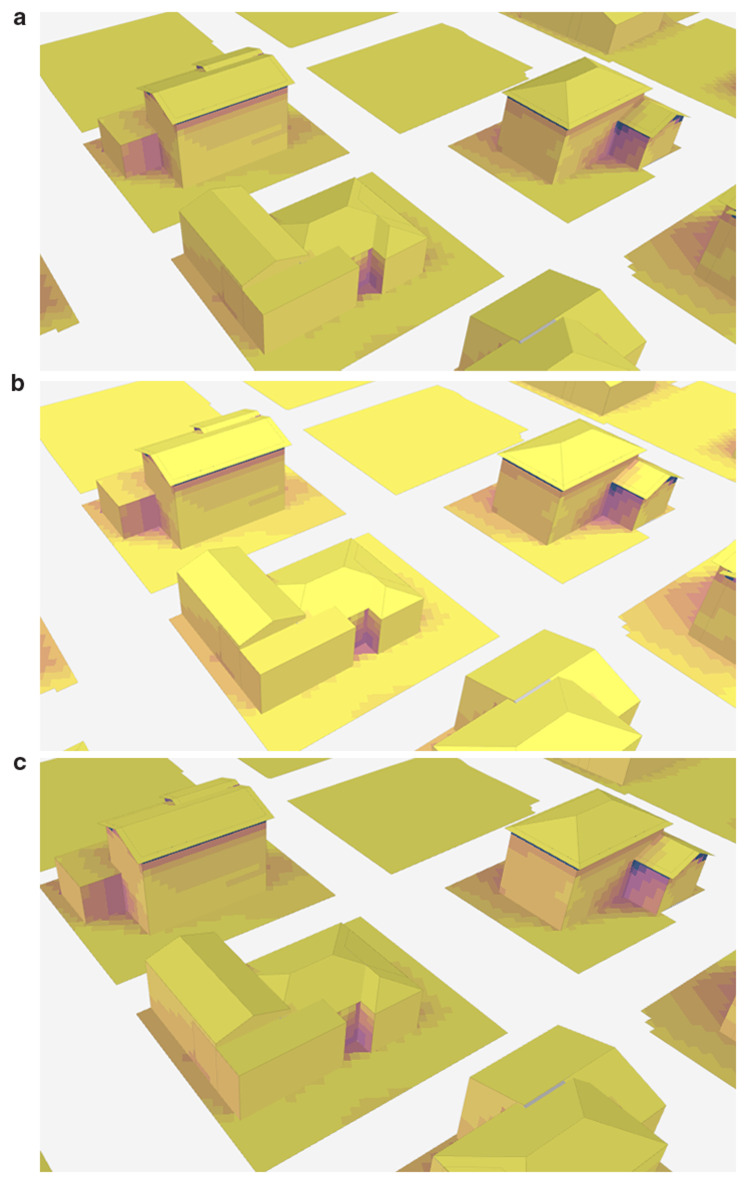
Change of shadow at different times on August 10th: ( **a**) 7.00, ( **b**) 14.00, and ( **c**) 20.00.

In order to examine the shadow change during one day, the shadow conditions at 07.00 (a), 14.00 (b), and 20.00 (c) on August 10th are examined as illustrated in Figure 5. During midday, buildings are more fully illuminated by the sun, leading to reduced shadow lengths. In the study, the average building height is six storeys, and no significant shadowing effects are anticipated.

**Figure 6.**
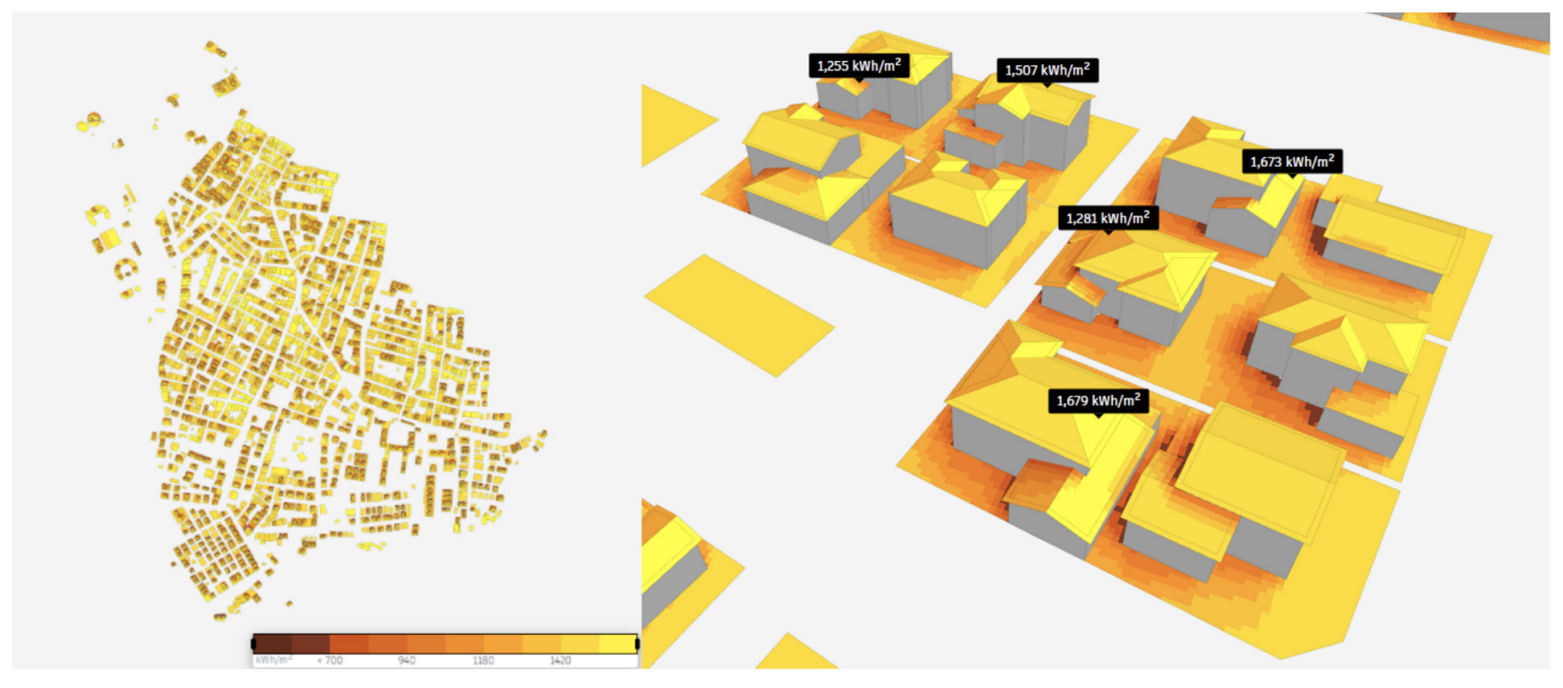
Results of the solar energy potential analysis.

In order to further explore the potential of PED, solar energy analysis is performed by extracting solar radiation data from the Copernicus database, scaling direct solar radiation for hourly sun angle assuming flat panels, and scaling diffuse solar radiation using the Isotropic sky model, as illustrated in Figure 6. The solar radiation incident on a part of the surface is briefly calculated by Equation 1. In this analysis, direct solar radiation is not included for hours when a surface is in the shade, but diffuse solar radiation is included for all hours. Finally, the photovoltaic output is obtained by entering panel efficiency and roof coverage information in the analysis (Bakkeli, H. (2024). Introduction to the solar energy analysis. https://help.autodeskforma.com/en/articles/8281900). In this study, efficiency is taken as 15% based on the fact that the efficiency of most solar panels is between 15% and 20% (Vourvoulias, A. (2014,). How efficient are solar panels? https://www.greenmatch.co.uk/blog/2014/11/how-efficient-are-solar-panels). Finally, in the study, the surface coverage parameter, which expresses the percentage of surfaces covered by productive solar panels excluding the supporting infrastructure, was taken as 60%, and analyses were conducted.

TotalIncidentRadiation=(SunResults∗DirectSolarRadiation)+DiffuseSolarRadiation(1)

According to the results of the analysis, the annual solar energy potential of the study region was reached. The total solar energy was 629,000,000 kWh, and the average solar energy was 1,410 kWh/m ^2^. The solar energy potentials of sample buildings in the study region are shown in Figure 6. It is observed that the solar energy potential is high on the roofs and different facades of the building as well.

In order to validate the results of the solar potential analysis, the PVGIS platform was utilized. According to the achieved results, an annual in-plane irradiation rate was 1,724.35 kWh/m². The analysis showed that fixed-angle photovoltaic (PV) systems generate the highest energy output in July and the lowest in December that is also in line with Autodesk Forma. A notable difference of 22.29% was identified between the solar potential analyses conducted on the Autodesk Forma and PVGIS platforms. This discrepancy is presumed to arise from the Autodesk Forma platform's reliance on a 3D building model for its assessments, whereas the PVGIS platform is limited to conducting evaluations based purely on geographical location.

Further analyses were conducted using auxiliary databases. The buildings in the Caferağa district were evaluated for their potential to install solar panels, taking into account structural conditions that may restrict their use. Approximately 69.4% of these buildings were built before 1990. Therefore, it is crucial to identify and implement alternative solutions for integrating solar energy into these aging structures, such as using heat pumps. To enhance the outcomes, the possibility of improving the detail level of the 3D model through the integration of CityGML and BIM could be explored. This could lead to a more refined and effective analyse and representation. Additionally, obtaining more detailed atmospheric and regional 2D and 3D data will increase the accuracy and reliability of assessments related to solar energy potential.

## Conclusion

In this study, a 3D spatial frame-work is designed and tested to examine the solar energy potential for PEDs in densely populated urban areas, where various constraints exist for utilizing other renewable resources. In this context, a comprehensive analysis of seasonal sunshine duration was undertaken, along with a careful examination of shadow variations throughout the day. It examines the types of data required for solar potential analyses and the benefits of utilizing 3D data derived from these sources. Within this data-driven study, several factors such as sun exposure duration and shadow change was identified as influencing factors of solar energy production. In order to analyze and validate this, several methods were deployed and compared. The developed concepts are validated in a PED district of Kadıkoy- Türkiye , where the study includes assessments of sunlight exposure, shading effects, and the overall solar energy potential deploying 3D GIS.

The results suggest that 3D spatial analyses could significantly enhance the potential for solar energy and provide valuable support to decision-makers during the complex processes of designing and expanding to borders of PEDs.

## Ethics and consent

Ethical approval and consent were not required.

## Data Availability

Zenodo: Exploring the sustainable energy Potential of Positive Energy Districts (PED) via Geographic Information System (GIS), https://doi.org/10.5281/zenodo.14604612 [ Demirel *et al.*, 2025] *This projects contains the following underlying data:* - *[buildings_caferaga_osm.cpg] (The provided data includes information about buildings in the Caferağa district of Kadikoy, Istanbul- Türkiye. All components of the sample dataset needs to be downloaded to function correctly.)* - *[buildings_caferaga_osm.dbf] (The provided data includes information about buildings in the Caferağa district of Kadikoy, Istanbul- Türkiye. All components of the sample dataset needs to be downloaded to function correctly.)* - *[buildings_caferaga_osm.prj] (The provided data includes information about buildings in the Caferağa district of Kadikoy, Istanbul- Türkiye. All components of the sample dataset needs to be downloaded to function correctly.)* - *[buildings_caferaga_osm.qmd] (The provided data includes information about buildings in the Caferağa district of Kadikoy, Istanbul- Türkiye. All components of the sample dataset needs to be downloaded to function correctly.)* - *[buildings_caferaga_osm.shp] (The provided data includes information about buildings in the Caferağa district of Kadikoy, Istanbul- Türkiye. All components of the sample dataset needs to be downloaded to function correctly.)* - *[buildings_caferaga_osm.shx] (The provided data includes information about buildings in the Caferağa district of Kadikoy, Istanbul- Türkiye. All components of the sample dataset needs to be downloaded to function correctly.)* - *[buildings_caferaga_osm.csv] (The provided data includes information about buildings in the Caferağa district of Kadikoy, Istanbul- Türkiye.)* *The study utilizes open-source data from Open Street Map (OSM).* Data is available under Creative Commons Attribution 4.0 International (CC BY 4.0 license).

## Software availability statement

The ESRI/ArcGIS City Engine platform is a proprietary software, where several alternatives exists as open source such as Blender, QGIS and CesiumJS for performing the transformation (Esri. (n.d.). ArcGIS City Engine. https://www.esri.com/en-us/arcgis/products/arcgis-cityengine/overview; Blender. (n.d.). Blender. https://www.blender.org/; QGIS. (n.d.). QGIS. https://www.qgis.org/; Cesium JS. (2023). Cesium JS. https://cesium.com/platform/cesiumjs/).

The AutoDesk Forma software is a proprietary software. As an alternative, Python and the Pysolar and pvlib libraries and CesiumJS software can be used to exploit the solar potential (Cesium JS. (2023).

Cesium JS. https://cesium.com/platform/cesiumjs/;

Autodesk. (2024). Autodesk Forma. https://www.autodesk.com/tr/products/forma/overview?term=1-YEAR&tab=subscription;

Python. (2024). Python. https://www.python.org/;

Pysolar. (n.d.). Pysolar. https://pysolar.org/;

Pylib. (2022). Pylib. https://pypi.org/project/pylib-general/).

Ladybug Tools. (2022). Ladybug Tools. https://www.ladybug.tools/;

Grasshopper. (2025). Ladybug Tools. https://www.grasshopper3d.com/group/ladybug;

Photovoltaic Geographical Information System. (2024). PVGIS. https://re.jrc.ec.europa.eu/pvg_tools/en/;

Rhino (2025). Ladybug Tools. https://www.food4rhino.com/en/app/ladybug-tools.
