# Supplementary material for: Evaluating the solar energy potential for Positive Energy Districts (PED) through advanced 3D Geographic Information System (GIS) analysis
Source: Open Res Eur. 2025 Jul 14;5:81. Originally published 2025 Mar 26. [Version 2] doi: 10.12688/openreseurope.19075.2 (PMC12365597; doi:10.12688/openreseurope.19075.2)
Supplement: Peer review history

# Reviewer report: Reviewer response for version 2

**DOI:** 10.21956/openreseurope.22472.r56859

**Published:** 2025-08-19

**Authors:** Sassi Rekik

**Affiliations:** 1 University of Monastir, Monastir, Tunisia

- The paper shows strong technical skills and presents a clear GIS-based framework to evaluate solar energy potential in urban Positive Energy District settings.
- Authors successfully combined AutoDesk Forma, Ladybug Tools, and PVGIS to check the accuracy of the results and uses open-source data.
- The methods are clearly explained, supported by helpful figures, and demonstrated through a well-chosen case study in Kadıkoy, Turkey.
- The use of 3D modeling improves the quality of spatial analysis, and the study offers valuable insights for improving decision-making in sustainable urban energy planning.

Therefore, I would confidently recommend the manuscript for indexing.

Is the study design appropriate and does the work have academic merit?

Yes

Is the work clearly and accurately presented and does it cite the current literature?

Yes

If applicable, is the statistical analysis and its interpretation appropriate?

Yes

Are all the source data underlying the results available to ensure full reproducibility?

Yes

Are the conclusions drawn adequately supported by the results?

Yes

Are sufficient details of methods and analysis provided to allow replication by others?

Yes

Reviewer Expertise:

Renewable energy planningGeographic Information Systems (GIS)Multi-Criteria Decision Making (MCDM)Machine Learning and Deep Learning applications in energy and spatial analysis

I confirm that I have read this submission and believe that I have an appropriate level of expertise to confirm that it is of an acceptable scientific standard.

# Reviewer report: Reviewer response for version 2

**DOI:** 10.21956/openreseurope.22472.r56852

**Published:** 2025-08-19

**Authors:** Mert Kompil

**Affiliations:** 1 European Commission, Joint Research Centre (JRC), Ispra, Italy

The paper focuses on Positive Energy Districts (PED) which aim to transform urban areas into carbon-neutral and zero-energy communities. It introduces a three-dimensional (3D) GIS-based framework designed to improve decision-making in PEDs, with a particular focus on spatiotemporal analyses of solar energy potential.

 The results indicate that 3D spatial analyses promote the potential for solar energy and aid decision-makers in the complex processes of designing and expanding PEDs. This is shown in a case study area – the Caferağa neighborhood of Kadıköy, Türkiye.

 Overall, the paper is well designed and written (some small typos / errors are supposed to be fixed). Previous revisions improved the text and linkages between the literature review and the rest of the paper. Strong empirical findings and methodological discussions would guide other studies in the field of 3D GIS-based spatial analysis of PED in urban areas. The paper is suggested to be indexed at Open Research Europe.

Is the study design appropriate and does the work have academic merit?

Yes

Is the work clearly and accurately presented and does it cite the current literature?

Yes

If applicable, is the statistical analysis and its interpretation appropriate?

Yes

Are all the source data underlying the results available to ensure full reproducibility?

Yes

Are the conclusions drawn adequately supported by the results?

Yes

Are sufficient details of methods and analysis provided to allow replication by others?

Yes

Reviewer Expertise:

geospatial analysis; urban policy; transport policy and research

I confirm that I have read this submission and believe that I have an appropriate level of expertise to confirm that it is of an acceptable scientific standard.

# Reviewer report: Reviewer response for version 1

**DOI:** 10.21956/openreseurope.20640.r52555

**Published:** 2025-04-18

**Authors:** Rovick Tarife

**Affiliations:** 1 Electrical Engineering, Mindanao State University - Iligan Institute of Technology, Iligan, Lanao del Norte, Philippines

1. While the paper demonstrates technical competence, it does not clearly distinguish itself from existing studies on PEDs and GIS-based solar analysis. The authors should explicitly articulate what is innovative about their methodology, case study application, or results compared to previous efforts such as those in the MAKING-CITY or POCITYF projects.

 2. The abstract should succinctly summarize the methodology, highlight the most important numerical results (e.g., energy potential in kWh/m²), and reflect your study’s unique contribution. Similarly, the conclusion would benefit from restructuring—currently, it reads more like an extended discussion. Consider separating key findings into a dedicated *Discussion* section and reserving the *Conclusion* for brief, impactful takeaways.

 3. The results are largely descriptive. To enrich the academic value, please include interpretative insights—why do certain facades outperform others? How do shading, orientation, and building geometry influence results? A discussion on implications for policy or retrofit strategies would also be welcome.

 4. The reference list is somewhat dated. Please consider incorporating recent scholarly publications and advancements in digital twin technologies, AI-based solar modeling, or urban energy optimization tools that are shaping the state-of-the-art in PED planning.

 5. The manuscript includes some grammatical and syntactical inconsistencies. A thorough language edit will greatly enhance readability. Reorganizing certain repetitive sections could also improve narrative flow.

 6. While the use of AutoDesk Forma and ArcGIS CityEngine is appropriate, validating your results using additional modeling platforms (e.g., PVGIS, EnergyPlus, or Ladybug Tools) or conducting a brief sensitivity analysis could strengthen the technical robustness.

 The paper has strong potential for publication once the above revisions are addressed. The subject matter is highly relevant, and the case study is well-chosen.

Is the study design appropriate and does the work have academic merit?

Yes

Is the work clearly and accurately presented and does it cite the current literature?

Partly

If applicable, is the statistical analysis and its interpretation appropriate?

Not applicable

Are all the source data underlying the results available to ensure full reproducibility?

Yes

Are the conclusions drawn adequately supported by the results?

Partly

Are sufficient details of methods and analysis provided to allow replication by others?

Yes

Reviewer Expertise:

Power System Optimization and Modelling, GIS Applications in Renewable Energy, Energy Systems Simulation, Microgrids

I confirm that I have read this submission and believe that I have an appropriate level of expertise to confirm that it is of an acceptable scientific standard, however I have significant reservations, as outlined above.

# Reviewer report: Reviewer response for version 1

**DOI:** 10.21956/openreseurope.20640.r52557

**Published:** 2025-04-04

**Authors:** Gokhan Sahin

**Affiliations:** 1 Utrecht University, Utrecht, The Netherlands

Comments to the Author This research “Exploring the sustainable energy Potential of Positive Energy Districts (PED) via Geographic Information System (GIS)”. I recommend this paper for publication if the requested items are modified.

 1. The difference of this study from the same studies in the literature is not clear. There are some simulation results, but the cause-effect link is not defined in the whole paper.

 2. The abstract should outline the findings of the proposed research study. It would be better if a table of articles that have been studied in the literature is included in the abstract.

 3. There are too many explanations in the conclusion section, it would be better if some of the explanations of the conclusion are written as a discussion section. The conclusion should be revised to be shorter and reflect the findings of the proposed research study.

 4. Finally, there are a few grammatical and syntax errors that need to be corrected.

 5. Reference sections should be updated. It would be better if the references are increased. There are too few references.

Is the study design appropriate and does the work have academic merit?

Yes

Is the work clearly and accurately presented and does it cite the current literature?

Partly

If applicable, is the statistical analysis and its interpretation appropriate?

No

Are all the source data underlying the results available to ensure full reproducibility?

Yes

Are the conclusions drawn adequately supported by the results?

No

Are sufficient details of methods and analysis provided to allow replication by others?

Yes

Reviewer Expertise:

NA

I confirm that I have read this submission and believe that I have an appropriate level of expertise to confirm that it is of an acceptable scientific standard, however I have significant reservations, as outlined above.
